# Dietary Effects of School-Based SNAP-Ed Education with and without Policy, Systems, and Environmental Change Strategies

**DOI:** 10.1007/s10900-025-01507-0

**Published:** 2025-08-19

**Authors:** Amanda Linares, Ramsha Baig, Sridharshi C. Hewawitharana, Gail Woodward-Lopez, Miranda Westfall Brown

**Affiliations:** Nutrition Policy Institute, University of California Agriculture and Natural Resources, 1111 Franklin Street, Oakland, CA 94607 USA

**Keywords:** Nutrition, Physical activity, School-based interventions, SNAP-Ed

## Abstract

There is potential value in combining education with policy, systems, and environmental (PSE) change strategies in school-based dietary and physical activity (PA) interventions. We investigated the impact of different combinations of Supplemental Nutrition Assistance Program-Education (SNAP-Ed) interventions on diet and PA and determined if student and school characteristics modified these impacts. A quasi-experimental, two-group (intervention (I) and comparison (C)), pre-post design examined the impact of interventions on diet and PA of 4th /5th grade students (n_I_=2,115;n_C_=1,102) in SNAP-Ed-eligible California public schools (n_I_=51;n_C_=18). Compared to students receiving no intervention, students receiving education + PSE decreased soda intake by 0.08 times/day [95% CI: −0.15, −0.02], increased whole fruit intake by 0.17 times/day [95% CI: 0.03–0.32], and increased total vegetable intake by 0.46 times/day [95% CI: 0.18–0.75]. Among students who received education only, sport and energy drink intake increased by 0.11 times/day [95% CI: 0.03–0.19] and 0.05 times/day [95% CI: 0.01–1.10] respectively, compared to students receiving no intervention, and fruit juice intake increased by 0.11 times/day [95% CI: 0.03, 0.20]. School urbanicity and Free and Reduced-Price Meal (FRPM) eligibility level modified intervention impacts. Compared to students from no intervention schools, students in urban schools receiving education only increased intake frequency of sweetened fruit drinks (β_interaction_[95% CI] = 0.12[0.01–0.23], and students from schools with FRPM above the state average receiving education + PSE significantly increased intake frequency of beans (β_interaction_[95% CI] = 0.14[0.04–0.24]. Findings highlight the benefit of coupling nutrition education with PSE changes and identify key areas for refining dietary and PA interventions in schools.

**Supplementary Information:**

The online version contains supplementary material available at 10.1007/s10900-025-01507-0.

## Introduction

Children in the United States are falling short of the nutrition and physical activity (PA) recommendations for maintaining good health [1–3]. Poor eating and activity habits developed early in life can translate to poor health status during adulthood including increased risk for overweight, obesity and chronic health problems [4]. Public health interventions can help address these shortcomings through various avenues, with school being an ideal place to intervene early in life. Nutrition and PA education, specifically in low-income schools, was traditionally a hallmark intervention and setting for the Supplemental Nutrition Assistance Program-Education (SNAP-Ed), or CalFresh Healthy Living (CFHL) as it is referred to in California. However, in 2010, the passing of the Healthy, Hunger Free Kids Act made policy, systems, and environmental (PSE) change approaches allowable, thus bringing this strategy to the forefront of CFHL interventions [5]. PSE strategies intend to make healthy choices easy, affordable, and the default option [6]. Additionally, PSE interventions can reach large population segments, and are often more cost effective and sustainable, making them an ideal complement to education-focused strategies [6].

Nutrition education-focused interventions in the school setting have been shown to be most effective when they include these critical components: (1) a multicomponent and multilevel approach, (2) adequate frequency and duration, (3) parental engagement, (4) age-appropriate, hands-on activities, (5) ensured fidelity of program delivery by training educators with a standardized protocol, (6) alignment between the objectives, intervention, and outcomes measured, and (7) incorporation of environmental changes [7]. The importance of multicomponent, multilevel approaches and the incorporation of environmental changes underscore the potential value of PSE approaches. In the school setting, PSE change interventions target: (1) policies, like school wellness or joint use policies, (2) practices, such as school procedures that could influence student nutrition and physical activity behavior, (3) systems, which often involve unwritten, ongoing, organizational changes that impact the whole school, and (4) the environment, which includes not only the built and physical environments, but also the economic, social, normative, or message environments [8]. Community partners, like CFHL, make the implementation of interventions with PSE strategies more feasible for schools by providing resources and support including funding, staffing, and subject matter expertise [9].

The California Department of Public Health (CDPH) implements CFHL through a network of 61 local health departments (LHDs). Schools are the most common setting for CDPH-CFHL intervention [10], thus making the LHD-school partnership critical to ensuring delivery of nutrition and PA-focused interventions to the students and families who need them most. While school-based CDPH-CFHL interventions have been shown to be effective at improving student diet and PA [11, 12], considering the unique student and school characteristics that exist throughout such a large and diverse state and tailoring interventions accordingly has the potential to increase their impact.

In response to the increasing emphasis on PSE and multicomponent interventions in CFHL, this study aims to investigate the impact of different combinations of intervention components on student dietary and PA behaviors. Furthermore, this study examines whether student and school characteristics modify the impact of intervention combinations on these behaviors.

## Methods

### School Recruitment and Study Methodology

This study utilized a quasi-experimental, two-group, pre-post design to examine the impact of various combinations of intervention types on student diet and PA. Participating schools were located throughout California, included fourth and fifth grades, and were eligible to receive CFHL programming, which is typically determined by the presence of at least 50% of students qualifying for Free and Reduced-Price Meals (FRPM). Data from 2017 to 2018 were used to determine CFHL eligibility. LHDs invited schools to join the study (i.e., evaluate their intervention) if they planned to partner with them on CDPH-CFHL programming during the 2021–2022 school year. The interventions included combinations of policy, systems, and environmental (PSE) changes, direct education (DE) (e.g., interactive, classroom-based education), and indirect education (IE) (e.g., newsletters or recipes). Intervention schools were classified into two groups: those exposed to interventions involving both PSE and education ([PSE, DE, and IE] or [PSE and DE]), and those exposed to education interventions alone (DE and IE, or DE only). The exposure to intervention components was determined using an online SNAP-Ed reporting system known as the Program Evaluation and Reporting System (PEARS) for the federal fiscal year (FFY) 21–22 [13]. Schools included as comparisons met the same CFHL eligibility criteria but had not partnered with any agency administering CFHL intervention in at least the last three school years and were selected from the same LHD jurisdictions as intervention schools. A final convenience sample of 51 intervention and 18 comparison schools agreed to participate. Comparison schools received a $1000 stipend upon completing all study requirements.

At least three classrooms of students at each school were administered voluntary surveys both before CDPH-CFHL intervention began (pre) and after intervention was complete (post). As mandated by a school or district, passive parental consent was obtained by distributing an opt-out form at least two weeks prior to survey administration. Students were provided with information about the survey prior to administration and were notified they could opt at any time during the study. Schools could select paper or online administration, and all surveys were administered by trained LHD nutrition educators and/or classroom teachers. Students were excluded from analyses if they were missing a pre-survey, a post-survey, or complete demographic information. Students were assigned unique ID numbers by survey administrators, and these IDs were used to match pre- and post-surveys.

Pre-survey data were collected September 2021 – March 2022 and post-survey data were collected March – June 2022. Though the intent was to complete data collection such that it represented a full school-year timeframe, securing class time for survey administration, especially post-COVID-19-related school closures, made this challenging. For this reason, the pre-to-post evaluation interval ranged from two to six months. Most schools (*n* = 60, 86%) conducted pre-surveys before winter/holiday break. This study was determined to be non-human subjects research by the Institutional Review Board at UC Davis and exempt by the State of California’s Committee for the Protection of Human Subjects.

### Collection of Student Dietary and Physical Activity Data

Students self-reported their dietary and PA behaviors using the Eating and Activity Tool for Students (EATS) (see supplemental material). As dietary questions ask about behaviors on the previous day and are meant to assess usual intake, study personnel were instructed to survey students on Tuesday-Friday and not following holidays or other days off from school.

Intake frequencies for fruits, vegetables, and beverages were assessed with 14 questions adapted from the validated School Physical Activity and Nutrition (SPAN) survey [14, 15]. Five questions assessed intake frequency of vegetables, with one question each for starchy vegetables (corn, non-fried potatoes, peas), orange vegetables, salad and green vegetables, other vegetables, and beans. Two questions assessed intake frequency of fruit (fruit, 100% fruit juice). Six questions assessed intake frequency of sugar-sweetened beverages (SSBs) (one each for fruit drinks, sports drinks, regular soda, energy drinks, sweetened coffee and tea, flavored milk). One question assessed intake frequency of water. With the exception of the fruit question, the four response options ranged from “*No*,* I didn’t eat/drink ___ yesterday”* to “*Yes*,* I ate/drank ____ 3 or more times yesterday.”* The fruit question had 5 response options ranging from “*No*,* I didn’t eat fruit yesterday”* to *“Yes*,* I ate fruit 5 or more times yesterday.”* Individual fruit, vegetable, and SSB responses were summed to obtain total fruit, total vegetable, and total SSB intake frequencies.

The survey also asked about two PA behaviors: (1) the number of days per week students were active for at least 60 min daily, to measure attainment of the moderate-to-vigorous PA (MVPA) recommendation [16] and (2) the number of days per week students had physical education (PE) class. The question assessing the number of days per week students were active for at least 60 min was used in its validated form [14, 15], and the PE-related question was developed by the researchers to evaluate specific programmatic priorities.

### Collection of Student and School-Level Demographic Data

Students self-reported their age, gender, race/ethnicity, and grade on the survey. School demographic data including total student enrollment, proportion of students eligible for FRPM, and grade levels served was retrieved from California Department of Education (CDE) data for the 2021-22 school year [17–19]. The proportion of students that were eligible for FRPM was divided into two categories based on the state average of proportion of FRPM-eligible students among CFHL-eligible schools in California (0.788). Schools that had a proportion of FRPM-eligible students greater than 0.788 were classified as ‘Schools above state average FRPM’, and the remainder were classified as ‘Schools at/below state average FRPM’. Urbanicity of schools was determined using the 2019 National Center for Education Statistics (NCES) Public School Locale data and categorized as ‘Urban’ or ‘Rural’ based on locale codes, a geographic indicator that identifies the type of area where a school is located [20].

### Statistical Analysis

The analytic sample was comprised of participants with complete pre- and post-test survey responses. Descriptive statistics were computed to summarize the socio-demographic characteristics of the study sample. To examine differences in school characteristics across intervention groups, we conducted Fisher’s exact tests for categorical variables and ANOVAs for continuous variables. Likewise, differences in student characteristics between intervention categories were assessed using Chi-square tests for categorical variables and F-tests for continuous variables, while accounting for clustering by schools.

Generalized estimating equations (GEE) were used to examine the impact of intervention combinations on the differences in change between intervention and comparison groups for dietary (water, SSBs, fruit and vegetable intake frequencies) and PA (days of achieving MVPA for at least 60 min and days of PE) behaviors, while accounting for clustering by schools and adjusting for outcome at pre-test, student’s age, gender, and race/ethnicity, and school urbanicity, grade span, FRPM eligibility, and total enrollment. In addition to this, standardized effect sizes (ES) were calculated using Hedge’s approach, dividing the estimated intervention effect by the pooled within-cluster standard deviation. ES were interpreted using Cohen’s (1988) guidelines, where 0.2 is small, 0.5 medium, and 0.8 large [21, 22]. Generalized linear models were used to determine if school and student characteristics modified the impact of intervention groups on behavioral outcomes. All analyses were performed in SAS 9.4 (SAS Institute Inc., Cary, NC, USA). Statistical significance was set to a p-value of < 0.05.

## Results

### Study Sample

After excluding study participants due to missing a pre-survey (*n* = 547), a post-survey (*n* = 1517), all pre- and post-survey responses (*n* = 12), or any missing (*n* = 2) or discrepancies (*n* = 995) in demographic information, the final analytic sample comprised 3,205 students and 69 schools. Of those, 26 schools and 1,072 students were exposed to education with PSE interventions, 25 schools and 1,033 students were exposed to education interventions only, and the remaining 18 schools and 1,100 students were not exposed to any intervention (Table 1). The majority of schools were elementary and located in urban areas with similar proportions across exposure groups (*p* = 0.79 and *p* = 0.06, respectively for school grade span and urbanicity). Approximately half of the schools had above the state average proportion of FRPM-eligible students and were comparable between exposure categories (58% education + PSE vs. 40% education only vs. 61% comparison, *p* = 0.32). On average, the total student enrollment in schools exposed to education with PSE interventions, education only interventions, and comparison schools was 493, 518, and 510, respectively, which was not statistically different across exposure groups (*p* = 0.85). The mean age of the study participants was 9.7 years with an equal proportion of boys and girls which was similar across all exposure groups (*p* = 0.64 and *p* = 0.20, respectively). Almost half of the participants identified as Hispanic/Latino and approximately one fourth of students belonged to a multiracial group. There were no differences in racial/ethnic groups between exposure categories (*p* = 0.11). Approximately two-thirds of students were fourth graders.

**Table 1:** Socio-demographic characteristics of study sample, by intervention groups, federal fiscal year 2021–2022 (*n* = 3,205 students; 69 schools), California

	Total sample (*n* = 3205 students; 69 schools)	Intervention (*n* = 2105 students; 51 schools)		Comparison (*n* = 1100 students; 18 schools)	
		Education + PSE^a^ intervention (*n* = 1072 students; 26 schools)	Education only intervention (*n* = 1033 students; 25 schools)		
	*n* (%)	*n* (%)			*p*-value
**Student Characteristics**^**b, c**^					
*Race/Ethnicity*					
Asian	128 (4.0)	75 (7.0)	33 (3.2)	20 (1.8)	0.108
Black	134 (4.2)	52 (4.8)	33 (3.2)	49 (4.4)	
Latino	1683 (52.5)	546 (50.9)	590 (57.1)	547 (49.7)	
White	306 (9.6)	89 (8.3)	86 (8.3)	131 (11.9)	
Multiracial	924 (28.8)	295 (27.5)	283 (27.4)	346 (31.4)	
*Another race/ethnicity*	30 (0.9)	15 (1.4)	8 (0.8)	7 (0.6)	
Gender					
Female	1622 (51.8)	559 (53.3)	500 (49.4)	563 (52.7)	0.198
Male	1509 (48.2)	490 (46.7)	513 (50.6)	506 (47.3)	
*Grade*					
4th	1953 (60.9)	649 (60.5)	595 (57.6)	709 (64.4)	0.777
5th	1252 (39.1)	423 (39.5)	438 (42.4)	391 (35.6)	
	**Mean (SD)**	**Mean (SD)**			**p-value**
Age	9.7 (0.7)	9.7 (0.7)	9.7 (0.7)	9.6 (0.7)	0.637
**School Characteristics**^**d**^					
	**n (%)**	**n (%)**			**p-value**
*Urbanicity*					
Urban	58 (84.1)	25 (96.2)	20 (80.0)	13 (72.2)	0.065
Rural	11 (15.9)	1 (3.8)	5 (20.0)	5 (27.8)	
*FRPM level*^**e**^					
At/below state average FRPM	33 (47.8)	11 (42.3)	15 (60.0)	7 (38.9)	0.317
Above state average FRPM	36 (52.2)	15 (57.7)	10 (40.0)	11 (61.1)	
*Grade span*					
Preschool & elementary	7 (10.1)	2 (7.7)	4 (16.0)	1 (5.6)	0.788
Elementary	55 (79.7)	22 (84.6)	18 (72.0)	15 (83.3)	
Elementary & middle	7 (10.1)	2 (7.7)	3 (12.0)	2 (11.1)	
	**Mean (SD)**	**Mean (SD)**			**p-value**
Total enrollment	506.4 (156.7)	493.0 (171.8)	517 (145.0)	509.9 (156.9)	0.850

^a^Policy, systems, and environment

^b^Student characteristics were self-reported

^c^P-values derived from Chi-square test for categorical characteristics, and F-tests for continuous characteristics, and accounted for clustering by schools

^d^P-values derived from Fisher’s exact test for categorical characteristics, and ANOVA tests for continuous characteristics

^e^Proportion of students eligible for Free and Reduced-price Meals (FRPM) categorized as a binary variable based on state average proportion of students eligible for FRPM (0.788) among CalFresh Healthy Living-eligible schools.

### Intervention

Of 51 schools that implemented a CDPH-CFHL intervention, 26 schools (51%) implemented education with PSE interventions (PSE + DE + IE [*n* = 22] and PSE + DE [*n* = 4]), 25 schools (49%) implemented education only interventions (DE + IE [*n* = 8] and DE only [*n* = 17]). Of those who implemented at least one PSE strategy (26 schools), the top 3 PSE strategies were (1) PA facilities (65%, 17 schools) such as playground markings or stencils; (2) non-PE PA (58%, 15 schools) such as classroom breaks for PA; and (3) utilization of school gardens (58%, 15 schools) including initiation and maintenance, support for nutrition education, and produce distribution. The most used DE curriculum was Let’s Eat Healthy (39%, 20 schools), followed by CATCH (K-5) – Kids Club Manual and Activity Box (33%, 15 schools) [23, 24]. Among those who carried out IE activities (30 schools), the most common intervention topics were fruits and vegetables, MyPlate food groups, and portions for healthy eating patterns (63%, 19 schools).

### Intervention Impact

There was no statistically significant difference in the change in total SSB intake frequencies between either intervention group and the comparison group (Table 2). However, the frequency of regular soda intake among students whose schools implemented education with PSE interventions decreased by 0.08 times/day [95% CI: −0.15, −0.02; ES: 0.09] compared to those who were not exposed to any intervention. Conversely, in comparison with no intervention students, the frequency of sports and energy drink intakes increased by 0.11 times/day [95% CI: 0.03, 0.19; ES: 0.12] and 0.05 times/day [95% CI: 0.01, 1.10; ES: 0.08], respectively, among students where schools were exposed to education only interventions.

**Table 2:** Adjusted^a^ mean differences in change in dietary intake frequencies and physical activity behaviors among study participants, by intervention group, federal fiscal year 2021–2022 (*n* = 3,205 students, 69 schools), California

Outcomes	*n* students; *n* schools	Adjusted mean difference in change between education + PSE^b^ intervention and comparison students	Adjusted mean difference in change between education only intervention and comparison students				
		β (95% CI^c^)	*p*-value	Effect size^d^	β (95% CI)	*p*-value	Effect size^d^
Water	3100 students; 69 schools	−0.02 (−0.11, 0.07)	0.627	0.02	0.003 (−0.08, 0.08)	0.947	0.003
Sugar-sweetened beverages	3068 students; 69 schools	−0.12 (−0.38, 0.13)	0.354	0.04	0.09 (−0.15, 0.33)	0.472	0.03
Fruit drinks	3099 students; 69 schools	−0.02 (−0.10, 0.06)	0.698	0.02	0.03 (−0.06, 0.12)	0.568	0.03
Sports drinks	3105 students; 69 schools	0.02 (−0.06, 0.09)	0.649	0.02	0.11 (0.03, 0.19)	0.007*	0.12
Regular soda	3102 students; 69 schools	−0.08 (−0.15, −0.02)	0.013*	0.09	0.01 (−0.05, 0.07)	0.684	0.01
Energy drinks	3104 students; 69 schools	0.01 (−0.05, 0.06)	0.845	0.02	0.05 (0.01, 0.10)	0.019*	0.08
Sweetened tea/coffee	3110 students; 69 schools	0.02 (−0.04, 0.08)	0.551	0.02	−0.003 (−0.06, 0.06)	0.930	0.004
Flavored milk	3101 students; 69 schools	−0.08 (−0.17, 0.02)	0.120	0.08	−0.04 (−0.16, 0.08)	0.500	0.04
Fruit, including 100% fruit juice	3106 students; 69 schools	0.23 (0.03, 0.43)	0.024*	0.10	0.12 (−0.07, 0.31)	0.223	0.06
Fruit, excluding 100% fruit juice	3114 students; 69 schools	0.17 (0.03, 0.32)	0.016*	0.10	0.02 (−0.12, 0.16)	0.748	0.01
100% fruit juice	3114 students; 69 schools	0.06 (−0.04, 0.15)	0.232	0.05	0.11 (0.03, 0.20)	0.009*	0.10
Vegetables	3092 students; 69 schools	0.46 (0.18, 0.75)	0.002*	0.17	0.05 (−0.21, 0.31)	0.710	0.02
Starchy vegetables	3125 students; 69 schools	0.14 (0.05, 0.23)	0.003*	0.14	0.03 (−0.04, 0.10)	0.439	0.03
Orange vegetables	3116 students; 69 schools	0.09 (0.01, 0.17)	0.035*	0.09	0.03 (−0.04, 0.10)	0.461	0.03
Green vegetables	3114 students; 69 schools	0.12 (0.02, 0.23)	0.026*	0.11	0.01 (−0.09, 0.11)	0.847	0.01
Other vegetables	3116 students; 69 schools	0.15 (0.06, 0.25)	0.001*	0.14	0.005 (−0.09, 0.10)	0.922	0.005
Beans	3117 students; 69 schools	0.08 (0.001, 0.15)	0.047*	0.10	0.02 (−0.05, 0.10)	0.529	0.02
Days with 60 min + moderate-to-vigorous physical activity	3091 students; 69 schools	0.27 (−0.01, 0.54)	0.057	0.11	0.01 (−0.24, 0.26)	0.919	0.004
Days with physical education	3067 students; 69 schools	−0.01 (−0.50, 0.48)	0.969	0.01	−0.13 (−0.55, 0.28)	0.525	0.09

^a^Models adjusted for school total enrollment, Free and Reduced-price Meal eligibility, urbanicity, grade span; student self-reported age, gender, race/ethnicity, and outcome at pre-test and accounted for clustering by school

^b^Policy, systems, and environment

^c^Confidence interval

^d^Effect size calculated dividing intervention effect (β) by pooled within-cluster standard deviation

*Statistically significant, p-value < 0.05

For students from schools that implemented PSE with education interventions, there was a significant increase (relative to the comparison group) in frequency of intake of total fruit (including 100% fruit juice) by 0.23 times/day [95% CI: 0.03, 0.43; ES: 0.10], total vegetables (excluding beans) by 0.46 times/day [95% CI: 0.18, 0.75; ES: 0.17], and beans by 0.08 times/day [95% CI: 0.001, 0.15; ES: 0.10]. More specifically, intake of fruits (excluding 100% fruit juice) increased by 0.17 times/day [95% CI: 0.03, 0.32; ES: 0.10], starchy vegetables by 0.14 times/day [95% CI: 0.05, 0.23; ES: 0.14], orange vegetables by 0.09 times/day [95% CI: 0.01, 0.17; ES: 0.09], green vegetables by 0.12 times/day [95% CI: 0.02, 0.23; ES: 0.11], and other vegetables by 0.15 times/day [95% CI: 0.06, 0.25; ES: 0.14], compared to students from schools that did not implement any intervention (Table 2). Relative to schools not receiving intervention, among students from schools that implemented education only interventions, although there was no significant difference in total fruit intake frequency (including 100% fruit juice), there was a statistically significant increase in 100% fruit juice consumption by 0.11 times/day [95% CI: 0.03, 0.20; ES: 0.10].

There was no significant statistical change in the number of MVPA days last week among students whose schools implemented education with PSE interventions (β [95% CI]: 0.27 [−0.01, 0.54]; ES: 0.11) or education only interventions (β [95% CI]: 0.01 [−0.24, 0.26]; ES: 0.004) compared to those who were not exposed to any intervention (Table 2). Similarly, there was no substantial difference in the number of PE days last week among students whose schools implemented education with PSE interventions (β [95% CI]: 0.01 [−0.50, 0.48]; ES: 0.01) or education only interventions (β [95% CI]: −0.13 [−0.55, 0.28]; ES: 0.09) compared to those who did not implement any intervention (Table 2).

### Effect Modification By School and Student Characteristics On Intervention Groups and Dietary and Physical Activity Outcomes

Total enrollment, urbanicity, FRPM eligibility, and student gender statistically significantly modified the effect of the intervention on certain dietary outcomes, but not PA outcomes. (Table 3).

**Table 3:** Interaction effects between school characteristics and intervention groups on change in student’s dietary intake and physical activity outcomes among study participants, federal fiscal year 2021–2022 (*n* = 3,025 students; 69 schools), California

Outcomes	*n* students; *n* schools	School Total Enrollment^a^			Urbanicity^b^			FRPM level^c^		
		Interaction term between total enrollment and education + PSE^d^ intervention β (95% CI^e^)	Interaction term between total enrollment and education only intervention β (95% CI)	Type 3 *p*-value	Interaction term between urbanicity and education + PSE intervention β (95% CI)	Interaction term between urbanicity and education only intervention β (95% CI)	Type 3 *p*-value	Interaction term between FRPM and education + PSE intervention β (95% CI)	Interaction term between FRPM and education only intervention β (95% CI)	Type 3 *p*-value
Dietary intake (change in number of times in past day from pre-test to post-test)										
Water	3100 students; 69 schools	0.04 (−0.01, 0.09)	0.02 (−0.02, 0.05)	0.343	−0.10 (−0.27, 0.07)	−0.12 (−0.35, 0.10)	0.540	−0.07 (−0.28, 0.14)	−0.10 (−0.30, 0.09)	0.569
Sugar-sweetened beverages	3068 students; 69 schools	0.04 (−0.10, 0.18)	−0.02 (−0.17, 0.13)	0.708	0.19 (−0.46, 0.85)	−0.17 (−0.93, 0.59)	0.543	−0.44 (−0.95, 0.06)	−0.09 (−0.68, 0.50)	0.214
Fruit drinks	3099 students; 69 schools	0.02 (−0.03, 0.07)	0.02 (−0.04, 0.07)	0.751	−0.02 (−0.21, 0.16)	−0.30 (−0.51, −0.09)	0.015*	−0.11 (−0.25, 0.04)	0.07 (−0.14, 0.28)	0.113
Sports drinks	3105 students; 69 schools	−0.02 (−0.06, 0.01)	0.001 (−0.05, 0.05)	0.393	0.03 (−0.12, 0.18)	0.14 (−0.06, 0.34)	0.341	0.01 (−0.14, 0.16)	−0.01 (−0.21, 0.18)	0.970
Regular soda	3102 students; 69 schools	0.03 (−0.01, 0.08)	−0.01 (−0.06, 0.04)	0.041*	0.18 (0.03, 0.33)	0.02 (−0.16, 0.21)	0.582	−0.05 (−0.19, 0.08)	0.07 (−0.11, 0.25)	0.200
Energy drinks	3104 students; 69 schools	0.02 (0.0003, 0.04)	0.01 (−0.01, 0.03)	0.225	−0.03 (−0.14, 0.08)	0.05 (−0.08, 0.17)	0.445	−0.02 (−0.12, 0.07)	−0.07 (−0.17, 0.03)	0.434
Sweetened tea/coffee	3110 students; 69 schools	0.003 (−0.05, 0.05)	−0.01 (−0.06, 0.03)	0.607	−0.11 (−0.23, 0.01)	−0.05 (−0.20, 0.11)	0.542	−0.07 (−0.19, 0.05)	−0.14 (−0.29, 0.02)	0.270
Flavored milk	3101 students; 69 schools	−0.03 (−0.08, 0.02)	−0.02 (−0.08, 0.04)	0.542	−0.05 (−0.23, 0.13)	−0.09 (−0.32, 0.15)	0.749	−0.04 (−0.24, 0.16)	0.16 (−0.07, 0.39)	0.238
Fruit, including 100% fruit juice	3106 students; 69 schools	0.11 (−0.01, 0.23)	0.07 (−0.03 0.17)	0.231	−0.33 (−0.69, 0.02)	−0.13 (−0.64, 0.38)	0.540	−0.49 (−0.86, −0.12)	−0.33 (−0.86, 0.20)	0.054
Fruit, excluding 100% fruit juice	3114 students; 69 schools	0.06 (−0.03, 0.14)	0.01 (−0.06, 0.08)	0.326	−0.22 (−0.48, 0.04)	0.01 (−0.36, 0.38)	0.622	−0.30 (−0.57, −0.02)	−0.23 (−0.56, 0.11)	0.122
100% fruit juice	3114 students; 69 schools	0.04 (−0.01, 0.10)	0.06 (0.01, 0.11)	0.226	−0.05 (−0.22, 0.11)	−0.13 (−0.36, 0.09)	0.506	−0.16 (−0.33, 0.01)	−0.07 (−0.33, 0.18)	0.180
Vegetables	3092 students; 69 schools	0.13 (−0.06, 0.32)	0.10 (−0.04, 0.24)	0.307	−1.01 (−1.45, −0.57)	−0.65 (−1.28, −0.02)	0.122	−0.54 (−1.11, 0.04)	−0.23 (−0.91, 0.46)	0.190
Starchy vegetables	3125 students; 69 schools	0.05 (−0.01, 0.11)	0.03 (−0.01, 0.10)	0.316	−0.25 (−0.43, −0.08)	−0.08 (−0.29, 0.14)	0.472	−0.13 (−0.32, 0.06)	−0.07 (−0.28, 0.14)	0.394
Orange vegetables	3116 students; 69 schools	0.01 (−0.04, 0.06)	0.04 (−0.003, 0.08)	0.226	−0.20 (−0.34, −0.07)	−0.23 (−0.41, −0.05)	0.124	−0.05 (−0.23, 0.12)	0.04 (−0.16, 0.23)	0.563
Green vegetables	3114 students; 69 schools	0.06 (0.001, 0.13)	0.04 (−0.01, 0.09)	0.126	−0.08 (−0.25, 0.09)	−0.11 (−0.36, 0.14)	0.620	−0.25 (−0.45, −0.05)	−0.24 (−0.49, 0.01)	0.064
Other vegetables	3116 students; 69 schools	0.02 (−0.05, 0.09)	−0.01 (−0.07, 0.05)	0.536	−0.41 (−0.63, −0.19)	−0.13 (−0.41, 0.14)	0.416	−0.03 (−0.22, 0.17)	0.06 (−0.17, 0.29)	0.700
Beans	3117 students; 69 schools	0.004 (−0.04, 0.04)	0.02 (−0.01, 0.05)	0.403	0.168 (0.03, 0.31)	−0.08 (−0.28, 0.12)	0.457	−0.17 (−0.30, −0.05)	−0.05 (−0.22, 0.11)	0.037*
Physical activity (change in number of days in past week from pre-test to post-test)										
Days with 60 min + moderate-to-vigorous physical activity	3091 students; 69 schools	0.12 (−0.04, 0.28)	−0.04 (−0.17, 0.10)	0.140	−0.58 (−0.99, −0.16)	0.64 (−0.02, 1.30)	0.173	−0.23 (−0.78, 0.32)	−0.65 (−1.12, −0.17)	0.065
Days with physical education	3067 students; 69 schools	0.14 (−0.20, 0.48)	−0.11 (−0.45, 0.24)	0.089	−0.65 (−1.52, 0.23)	−0.36 (−1.38, 0.67)	0.531	−0.67 (−1.46, 0.12)	0.07 (−0.70, 0.84)	0.208

^a^Models adjusted for school urbanicity, FRPM level, grade span, student self-reported age, race/ethnicity, gender, outcome at baseline, and accounted for clustering by school. Total enrollment is scaled by 100 students.

^b^Models adjusted for school total enrollment, FRPM level, grade span, student self-reported age, race/ethnicity, gender, outcome at baseline, and accounted for clustering by school. Reference group of urbanicity is ‘urban schools’.

^c^Models adjusted for school total enrollment, urbanicity, grade span, student self-reported age, race/ethnicity, gender, outcome at baseline, and accounted for clustering by school. Proportion of students eligible for Free and Reduced-Price Meals (FRPM) categorized as a binary variable based on state average proportion of students eligible for FRPM among CalFresh Healthy Living-eligible schools. Reference group of FRPM level is ‘Above state average FRPM’.

^d^Policy, systems, and environment.

^e^Confidence interval.

*Statistically significant, p-value < 0.05.

There was a significant interaction between total enrollment and exposure groups on change in regular soda frequency consumption (education + PSE: β_interaction_[95% CI] = 0.03[−0.01, 0.08], education only: β_interaction_[95% CI]=−0.01[−0.06, 0.04], p-value: 0.04) (Table 3). However, when compared to students belonging to comparison schools, with every 100-unit increase in total student enrollment there was no significant difference in the change in frequency of regular soda intake among either intervention group (data not shown).

School urbanicity modified the effect of exposure groups on change in intake frequency of sweetened fruit drinks (p-value: 0.01) (Table 3); there was a significant increase in intake among students in urban schools exposed to education only interventions (but not education + PSE interventions) when compared to students from urban schools without any intervention (β_interaction_[95% CI] = 0.12[0.01, 0.23] (Table 4). Inversely, among students from rural schools exposed to education only interventions (but not education + PSE interventions), there was significant reduction of sweetened fruit drink intake compared to students from rural schools who were not exposed to any intervention (β_interaction_[95% CI]=−0.18[−0.35, −0.01] (Table 4).

**Table 4:** Adjusted mean difference in change in dietary intake frequencies between intervention and comparison students, stratified by statistically significant categorical effect modifiers among study participants, federal fiscal year 2021–2022 (*n* = 3,205 students; 69 schools), California

Outcomes	Effect modifiers		*n* students; *n* schools	Adjusted mean difference in change between education + PSE^a^ intervention and comparison students β (95% CI^b^)	*n* students; *n* schools	Adjusted mean difference in change between education only intervention and comparison students β (95% CI)
Dietary intake (change in number of times in past day from pre-test to post-test)						
Water	Student gender^c^	Female	559 students; 26 schools	−0.10 (−0.23, 0.02)	500 students; 25 schools	−0.04 (−0.15, 0.06)
		Male	490 students; 26 schools	0.07 (−0.02, 0.16)	513 students; 24 schools	0.06 (−0.03, 0.14)
Fruit drinks	Urbanicity^d^	Urban	1056 students; 25 schools	0.01 (−0.07, 0.09)	690 students; 20 schools	**0.12 (0.01**, 0.23)*
		Rural	16 students; 1 school	−0.01 (−0.17, 0.15)	343 students; 5 schools	**−0.18 (−0.35**, −0.01)*
Beans	FRPM level^e^	At/below state average FRPM	382 students; 15 schools	−0.03 (−0.12, 0.05)	447 students; 11 schools	−0.004 (−0.10, 0.09)
		Above state average FRPM	690 students; 10 schools	**0.14 (0.04**, 0.24)*	586 students; 15 schools	0.05 (−0.08, 0.17)

^a^Policy, systems, and environment

^b^Confidence interval

^c^Models adjusted for school total enrollment, school urbanicity, FRPM level, grade span, student self-reported age, race/ethnicity, outcome at baseline, and accounted for clustering by school.

^d^Models adjusted for school total enrollment, FRPM level, grade span, student self-reported age, race/ethnicity, gender, outcome at baseline, and accounted for clustering by school.

^e^Models adjusted for school total enrollment, urbanicity, grade span, student self-reported age, race/ethnicity, gender, school total enrollment, outcome at baseline, and accounted for clustering by school. Proportion of students eligible for free and reduced-price meals (FRPM) categorized as a binary variable based on state average proportion of students eligible for FRPM among CalFresh Healthy Living-eligible schools.

*Statistically significant, p-value < 0.05.

A statistically significant interaction was observed between school FRPM level and exposure groups for change in intake frequency of beans (p-value: 0.04) (Table 3), with a significant increase in bean intake among students from schools with FRPM above the state average (but not below the state average) that implemented PSE with education interventions compared to students who were unexposed (β_interaction_[95% CI] = 0.14[0.04, 0.24] (Table 4).

Although there was a significant interaction between student’s gender and exposure groups on the change in water intake (p-value: 0.03) (Table 5), there was no significant effect modification among males nor females with either intervention type when compared to students from comparison schools (Table 4).

**Table 5:** Interaction effects between student gender and intervention groups on change in student’s dietary intake and physical activity outcomes among study participants, federal fiscal year 2021–2022 (*n* = 3,025 students; 69 schools), California

Outcomes	*n* students; *n* schools	Student Gender^a^		
		Interaction term between gender and education + PSE^b^ intervention β (95% CI^c^)	Interaction term between gender and education only intervention β (95% CI)	*p*-value
Dietary intake (change in number of times in past day from pre-test to post-test)				
Water	3100 students; 69 schools	0.17 (0.05, 0.30)	0.10 (0.004, 0.21)	0.026*
*Sugar-sweetened beverages*	3068 students; 69 schools	−0.15 (−0.55, 0.26)	−0.26 (−0.69, 0.16)	0.444
Fruit drinks	3099 students; 69 schools	0.07 (−0.08, 0.22)	−0.04 (−0.18, 0.11)	0.436
Sports drinks	3105 students; 69 schools	−0.01 (−0.14, 0.12)	−0.02 (−0.16, 0.12)	0.965
Regular soda	3102 students; 69 schools	−0.06 (−0.19, 0.06)	−0.003 (−0.14, 0.14)	0.516
Energy drinks	3104 students; 69 schools	−0.02 (−0.08, 0.04)	−0.03 (−0.12, 0.05)	0.687
Sweetened tea/coffee	3110 students; 69 schools	−0.07 (−0.19, 0.06)	−0.08 (−0.19, 0.03)	0.319
Flavored milk	3101 students; 69 schools	−0.01 (−0.13, 0.11)	−0.05 (−0.17, 0.07)	0.733
*Fruit, including 100% fruit juice*	3106 students; 69 schools	0.14 (−0.17, 0.45)	−0.17 (−0.52, 0.17)	0.179
Fruit, excluding 100% fruit juice	3114 students; 69 schools	0.06 (−0.18, 0.29)	−0.07 (−0.31, 0.17)	0.541
100% fruit juice	3114 students; 69 schools	0.06 (−0.07, 0.19)	−0.10 (−0.26, 0.06)	0.176
*Vegetables*	3092 students; 69 schools	0.21 (−0.19, 0.61)	0.21 (−0.91, 0.62)	0.542
Starchy vegetables	3125 students; 69 schools	0.04 (−0.10, 0.19)	0.01 (−0.17, 0.19)	0.797
Orange vegetables	3116 students; 69 schools	0.09 (−0.05, 0.23)	0.06 (−0.08, 0.21)	0.461
Green vegetables	3114 students; 69 schools	0.01 (−0.17, 0.19)	0.06 (−0.12, 0.24)	0.760
Other vegetables	3116 students; 69 schools	0.06 (−0.07, 0.19)	0.04 (−0.10, 0.19)	0.658
*Beans*	3117 students; 69 schools	0.07 (−0.06, 0.20)	−0.01 (−0.12, 0.11)	0.499
Physical activity (change in number of days in past week from pre-test to post-test)				
Days with 60 min + moderate-to-vigorous physical activity	3091 students; 69 schools	−0.21 (−0.62, 0.21)	−0.29 (−0.64, 0.06)	0.302
Days with physical education	3067 students; 69 schools	−0.07 (−0.30, 0.16)	0.16 (−0.04, 0.36)	0.067

^a^Models adjusted for school total enrollment, FRPM level, grade span, urbanicity, student self-reported age, race/ethnicity, outcome at baseline, and accounted for clustering by school. Reference group of student gender is ‘Male students’.

^b^Policy, systems, and environment.

^C^Confidence interval.

*Statistically significant, p-value < 0.05.

## Discussion

Findings from this study reinforce the important role of PSE change strategies in comprehensive, multicomponent school-based nutrition education interventions for improving student dietary intake. However, no evidence was identified for the effectiveness of education with PSE nor education only, as implemented by the participating schools, for improving student PA outcomes.

Compared to students receiving no intervention, students receiving education with PSE saw increases in intake frequencies of whole fruit, total vegetables, and all vegetable subgroups. Students receiving education alone did not see any significant pre-post changes in intake frequency of fruits or vegetables. In this study, the most common nutrition-focused PSE intervention was the creation or maintenance of school gardens (*n* = 15 schools). A recent review of school-based interventions utilizing school garden activities demonstrated benefits to children’s nutrition knowledge, their attitudes about and acceptance of fruits and vegetables, and ultimately, behavior change with increased consumption of fruits and vegetables [25]. Another review identified six core strategies of garden programming that maximize its effectiveness and sustainability: (1) Hands on experiences (e.g., planting, harvesting), (2) preparing or cooking garden-grown fruits and vegetables, (3) accompanying garden/nutrition lessons, (4) gathering stakeholder input in the development, implementation, and maintenance, (5) involving parents and families, and (6) utilizing the produce harvested from the school garden [26]. CFHL-based garden interventions incorporate many of these strategies, including parent, stakeholder, and community involvement, hands-on work in the garden, utilizing garden produce for meals and/or snacks served onsite, and incorporating the garden into nutrition education (often with a specialized educator) [10]. Additionally, in the current program model, LHDs are encouraged to utilize garden-based approaches as a complement to other high-impact PSE strategies, such as implementing nutrition standards or increasing access to healthy foods [27]. In this sample, the majority of schools (80%) who engaged in garden-based activities also implemented other nutrition-related PSEs, including initiating or expanding farm-to-table or use of local produce (*n* = 6), improving cafeteria menus to include lighter fare (*n* = 6), and establishing new food distribution sites (*n* = 6). The results of this study reinforce the importance of complementing nutrition education with environmental strategies to make healthy foods accessible and appealing.

As it pertains to beverage consumption behaviors, compared to students receiving no intervention, students receiving education with PSE saw decreases in the intake frequency of soda, whereas students receiving education only saw increases in the intake frequency of sports and energy drinks and 100% fruit juice. There is research that mirrors some of these findings. A study by Sichieri et al. found that a multicomponent school-based intervention targeting SSB consumption was associated with decreased consumption of carbonated sweetened beverages but increased consumption of fruit juice [28]. One important factor in the current study is the presence of variation even within each intervention combination type. LHDs implementing CFHL can choose from a menu of education and PSE options to best fit their local needs. While they all must address key dietary and/or PA behaviors as defined by the SNAP-Ed program, not all of them target every behavior, nor are they carried out with the same intensity and/or for the same length of time. In fact, the decrease in soda consumption among students exposed to both education and PSE interventions was somewhat unexpected, given that the most common PSE change strategies were PA and garden-focused (i.e., likely not focused on beverage consumption behavior). It is important to note the lack of substantive changes observed in water consumption with both intervention combinations. This observation coupled with the others may indicate that nutrition curricula could benefit from a more robust focus on a broader array of healthy and unhealthy beverage types in education materials, and a need to reinforce PSE efforts addressing the availability and promotion of healthy SSB alternatives like water.

Although the effect sizes were small and the change in daily consumption frequencies may seem trivial among students receiving education with PSE, such incremental increases in consumption of fruits and vegetables and reductions in soda intake can accumulate over time, potentially resulting in meaningful gains at the weekly or monthly level. According to Cohen’s (1988) benchmarks, an effect size of less than 0.2 would be considered below the conventional threshold for a “small” effect; however, in social sciences research even small effects may have practical significance, especially when sustained over time [29].

Regarding effect modification, for total enrollment, urbanicity, FRPM eligibility, and student gender, significant interactions were identified. However, despite the interactions identified for school total enrollment and student gender, when compared to comparison schools, there was no significant difference by gender or school enrollment in the relative change in dietary intake frequencies among students from schools that implemented either intervention combination. For school urbanicity, there was a significant increase in intake of sweetened fruit drinks among students in urban schools and a significant decrease in intake among students in rural schools exposed to education interventions when compared to students from schools without intervention. The findings for rural schools should be interpreted with caution as there were a small number of rural schools, limiting our ability to generalize findings pertaining to school urbanicity. The increase in sweetened fruit drink intake observed in the more robust urban school sample, while unexpected, could indicate a need for stronger and perhaps specific beverage-focused content in the curricula options provided to CFHL implementers, and especially those working with youth in urban areas. A study utilizing school-based CDPH-CFHL dietary behavior data from the school year prior to this study indicated that SSB consumption behavior was not altered by the topically broad nutrition interventions delivered [12]. An Arizona study using a similar design to evaluate school-based SNAP-Ed programs found that general nutrition education was not enough to influence beverage-related behaviors, as consumption of SSBs similarly increased at post-test [30]. Among the two most popular curricula utilized in this study, Let’s Eat Healthy and CATCH, neither has a particularly strong focus on healthy beverage options. Given the availability and marketing of new beverages is ever-changing, and that children and adolescents may now be favoring non-soda SSBs [31], children may benefit from adopting and/or developing a CFHL allowable curriculum that focuses solely on healthy and unhealthy beverages.

There was a significant increase in intake of beans among students from schools where the proportion of students eligible for FRPM was above the state average and exposed to PSE interventions with education compared to students from schools without any intervention. While there is a chance this could be due to type 1 error, these findings suggest interventions combining education and PSE strategies could be more effective in supporting this healthy behavior among students in the highest need schools. This is promising for multiple reasons. Among all vegetable subgroups, school meal programs are least likely to provide the required servings of legumes [32]. While we do not know the proportion of student consumption of beans at school versus away from school, if this vegetable subgroup is improved by intervention, schools and school districts should take note when designing school menus. Additionally, beans are an affordable nutritional powerhouse. They are a good source of plant-based protein, high in fiber, are inexpensive when purchased dry, and are shelf-stable and convenient when canned [33]. These qualities make them an ideal component for healthy meals on a budget.

In this study, even though the most common PSE strategies implemented by intervention schools were focused on PA, we did not observe improvements in days per week of PE. This may reflect the nature of the PA interventions implemented, i.e., they targeted non-PE PA and PA facilities. We also did not observe improvements in student MVPA. Given this study was conducted in the school year immediately following COVID-19-related school closures, it is possible that normal, pre-COVID-19 school health and safety practices and procedures around PA and PE were not yet back in effect, potentially limiting student activity levels. Though limited, research assessing children’s sedentary and physical activity behaviors during the initial COVID-19 period and again a year later, indicates that though sedentary behaviors decreased from the onset to one-year, physical activity behaviors during the same period remained unchanged [34].

This study had several strengths and limitations. We included CFHL-eligible comparison schools in our quasi-experimental design, which allowed us to examine real world intervention impact. Given the uniqueness of CDPH-CFHL intervention delivery and the complexity of obtaining school buy-in, the intervention and comparison schools and classrooms were selected out of convenience rather than randomly assigned. This may have resulted in selection bias and lack of school-level representativeness. The interventions were self-selected by the schools in partnership with the LHDs. Although this contributes to their real-world relevance, findings are limited to the interventions that were selected and implemented by these schools. The survey used to assess student dietary and PA behaviors was validated with elementary students [15]. Despite this, students self-reported and therefore, data are subject to recall error and/or bias. To minimize any recall error, students were asked to report only dietary intake on the previous day and PA in the previous week. Yet, it is possible that the recall timeframes were not fully representative of all students’ usual intake and activity. Given California is a large and demographically and geographically diverse state, and our focus was CFHL-eligible schools, these findings may not be generalizable to other states and countries and higher income populations. Regarding effect modification, we identified one statistically significant result out of 17 outcomes tested. It is possible this is due to chance or low sample sizes, since this study was not originally designed to be powered to test for and detect these effects.

## Conclusion

Findings from this study support combining PSE strategies with nutrition education in the school setting to improve student fruit, vegetable, and SSB consumption behaviors. The findings also provide insight into ways current CDPH-CFHL interventions, many of which are employed broadly by SNAP-Ed in other states, could be refined and customized to improve effectiveness. For programs prioritizing SSBs, for example, education may be improved by including a strong and broad enough focus on individual SSB subtypes, and PSE interventions may benefit from more robust promotion of SSB alternatives like water. Gardens are already a popular area for PSE implementation in California schools. Our findings support the use of robust, multicomponent garden-based efforts as an ideal complement to CFHL core PSE strategies such as nutrition standards, behavioral economics, and access to healthy foods. Given the findings of effect modification by school characteristics, implementers should consider the unique characteristics and needs of each school and tailor interventions accordingly.

## Supplementary Information

Below is the link to the electronic supplementary material.

Supplementary Material 1

## Data Availability

The SNAP-Ed data, FFY 22 Program Evaluation and Reporting System data files, that support the findings of this study are not publicly available. The data are, however, available from the authors upon reasonable request and with the permission of the Nutrition Policy Institute (NPI). The SNAP-Ed data, FFY 22 Eating and Activity Tool for Students, cannot be shared publicly or privately as its institutional review board approval dictates. The secondary data sources used to generate school-level demographics are publicly available: Census Day Enrollment by School, 2019 (California Department of Education): https://www.cde.ca.gov/ds/ad/filesenrcensus.asp. Public Schools and Districts Data File, 2021 (California Department of Education): https://www.cde.ca.gov/ds/si/ds/pubschls.asp. Unduplicated Student Poverty- Free or Reduced-Price Meals Data (California Department of Education): https://www.cde.ca.gov/ds/ad/filessp.asp. National Center for Education Statistics. Public School File, 2019–2020 (US Department of Education): https://nces.ed.gov/programs/edge/Geographic/SchoolLocations.
