# Supplementary material for: Dietary Effects of School-Based SNAP-Ed Education with and without Policy, Systems, and Environmental Change Strategies
Source: J Community Health. 2025 Aug 19;51(1):24–36. doi: 10.1007/s10900-025-01507-0 (PMC12950050; doi:10.1007/s10900-025-01507-0)
Supplement: Supplementary file 1 — Supplementary Material 1 [file 10900_2025_1507_MOESM1_ESM.docx]

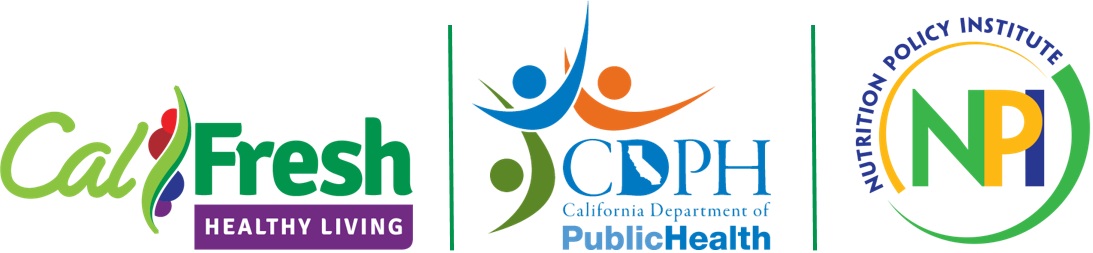


**Eating and Activity Tool for Students**

**(EATS)**

**Updated July 2020**

Created by the University of California Nutrition Policy Institute for the California Department of Public Health; funded by the United States Department of Agriculture Supplemental Nutrition Assistance Program- USDA SNAP, an equal opportunity provider and employer.

Parts of this survey were adapted from the School Physical Activity and Nutrition Project (SPAN – University of Texas, Austin). For source information about individual survey questions, contact Nutrition Policy Institute, Amanda Linares, amlinares@ucanr.edu.

**To be completed by LHD or school site**

Site Name or PEARS Site ID: __________________

Classroom (teacher): ________________________

**Directions:** This is a survey about what you eat and drink and your physical activity. For each question, either fill in the bubble (O) of the one best answer, or the box (□) for each true answer.

**Date: _______________**

**ID number: ­­­______________**

**1. How old are you?**

O 7

O 8

O 9

O 10

O 11

O 12

O 13

O 14

**2. What grade are you in?**

O 4^th^

O 5^th^

**3. Are you a boy or a girl?**

O Boy

O Girl

O I don't want to answer

**4. How do you describe yourself?**

**Choose all the boxes (□) that best describe you.**

□ American Indian or Alaska Native

□ Asian

□ Black or African American

□ Latino or Hispanic (Mexican, Salvadoran, Guatemalan, etc.)

□ Native Hawaiian or other Pacific Islander

□ White

□ Other: ___________________________

**5.** **How did you attend school yesterday?**

O In person

O Distance learning

O In person and distance learning

O I did not attend school yesterday in person or by distance learning

The next questions are about **what you ate or drank yesterday.**

**6. For lunch yesterday…**

O I ate the school lunch **at school**

O I ate the school lunch **at home**

O I did not eat the school lunch

**7. For breakfast yesterday…**

O I ate the school breakfast **at school**

O I ate the school breakfast **at home**

O I did not eat the school breakfast

**8. Yesterday, did you eat any potatoes, corn, or peas?**

**Do not count French fries or chips or sweet potatoes.**


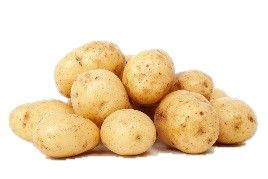

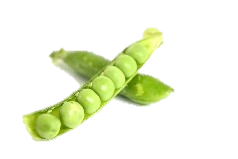

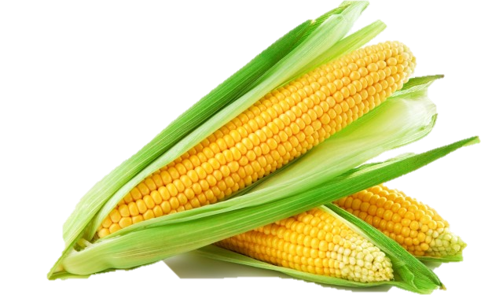


O No, I didn’t eat any of these vegetables yesterday.

O Yes, I ate these vegetables 1 time yesterday.

O Yes, I ate these vegetables 2 times yesterday.

O Yes, I ate these vegetables 3 or more times yesterday.

**9. Yesterday, did you eat any orange vegetables like:**

Carrots Sweet potatoes

Squash Other orange vegetables


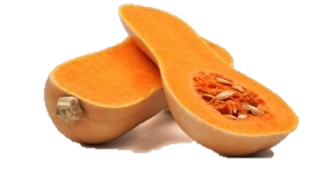

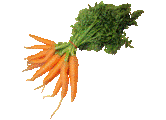

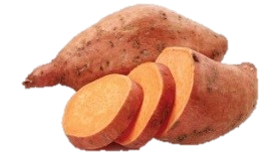


O No, I didn’t eat any orange vegetables yesterday.

O Yes, I ate orange vegetables 1 time yesterday.

O Yes, I ate orange vegetables 2 times yesterday.

O Yes, I ate orange vegetables 3 or more times yesterday.

**10. Yesterday, did you eat any salad or green vegetables like:**

Salad made with lettuce Spinach


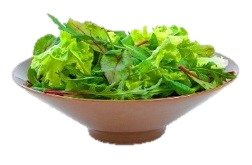

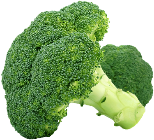

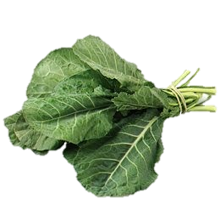

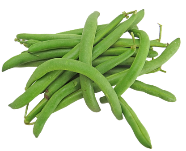


Broccoli Collard greens

Green beans Other greens

O No, I didn’t eat any salad or green vegetables yesterday.

O Yes, I ate salad or green vegetables 1 time yesterday.

O Yes, I ate salad or green vegetables 2 times yesterday.

O Yes, I ate salad or green vegetables 3 or more times yesterday.

**11. Yesterday, did you eat any other vegetables like:**


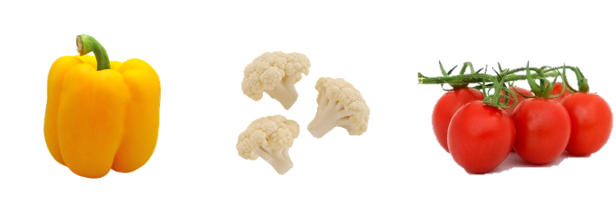
 Peppers Cucumbers

Tomatoes Mushrooms

Zucchini Eggplant

Artichokes Asparagus

Celery Cauliflower

Cabbage Other vegetables

O No, I didn’t eat any other vegetables yesterday.

O Yes, I ate other vegetables 1 time yesterday.

O Yes, I ate other vegetables 2 times yesterday.

O Yes, I ate other vegetables 3 or more times yesterday.

**12. Yesterday, did you eat any beans like:**

**
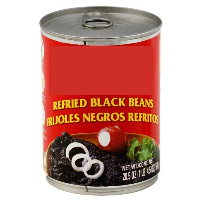
** Pinto beans Refried beans

Baked beans Other beans

**Do not count green beans.**

O No, I didn’t eat any beans yesterday.

O Yes, I ate beans 1 time yesterday.

O Yes, I ate beans 2 times yesterday.

O Yes, I ate beans 3 or more times yesterday.

**13. Yesterday, did you eat any fruit like:**

Fresh fruit Canned fruit


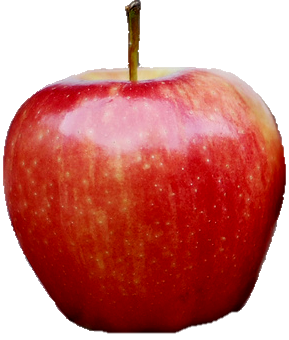

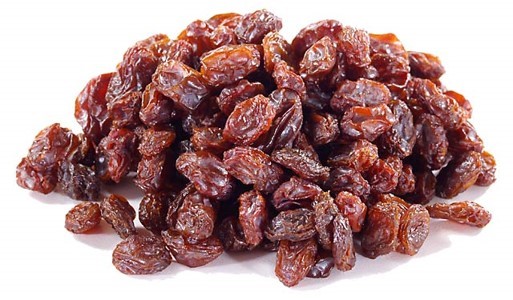

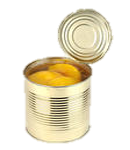


Frozen fruit Dried fruit

**Do not count fruit juice**.

O No, I didn’t eat any fruit yesterday.

O Yes, I ate fruit 1 time yesterday.

O Yes, I ate fruit 2 times yesterday.

O Yes, I ate fruit 3 times yesterday.

O Yes, I ate fruit 4 times yesterday.

O Yes, I ate fruit 5 or more times yesterday.


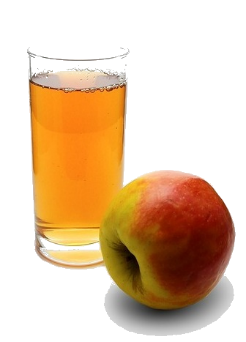
**14. Yesterday, did you drink any 100% fruit juice like:**

Orange juice Grape juice

Apple juice Other 100% juice

**Do not count** **punch, sports drinks, or other fruit-flavored drinks.**

O No, I didn’t drink any fruit juice yesterday.

O Yes, I drank fruit juice 1 time yesterday.

O Yes, I drank fruit juice 2 times yesterday.

O Yes, I drank fruit juice 3 or more times yesterday.

**15. Yesterday, did you eat any French fries or chips like:**


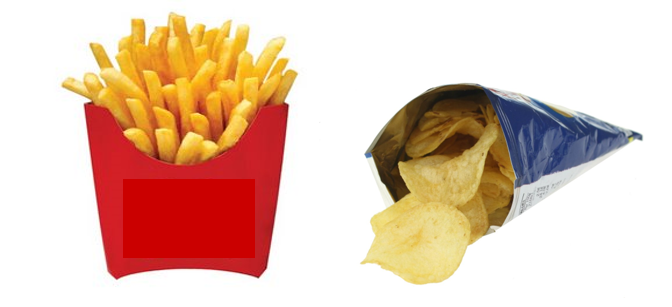
 Tortilla chips Cheese puffs

Potato chips Other chips

O No, I didn’t eat any French fries or chips yesterday.

O Yes, I ate French fries or chips 1 time yesterday.

O Yes, I ate French fries or chips 2 times yesterday.

O Yes, I ate French fries or chips 3 or more times yesterday.


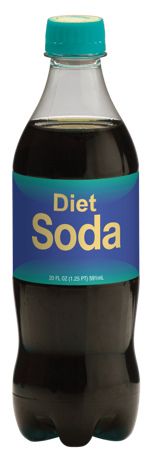


**16. Yesterday, did you drink any diet soda like:**

Diet cola Diet root beer

Diet lemon-lime soda Other diet soda

O No, I didn’t drink any diet soda yesterday.

O Yes, I drank diet soda 1 time yesterday.

O Yes, I drank diet soda 2 times yesterday.

O Yes, I drank diet soda 3 or more times yesterday.

For the questions below, **do not include any diet or unsweetened drinks.**

**17. Yesterday, did you drink any fruit drinks like:**

Punch Sweetened vitamin water


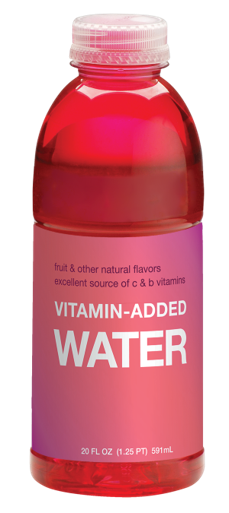

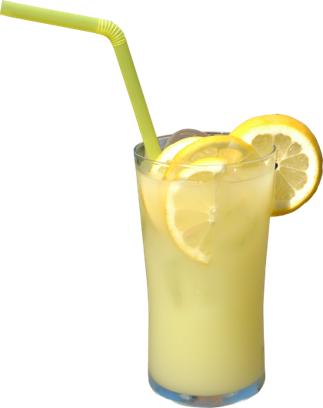


Agua fresca Lemonade

Other fruit-flavored drinks

**Do not count 100% fruit juice.**

O No, I didn’t drink any fruit drinks yesterday.

O Yes, I drank fruit drinks 1 time yesterday.

O Yes, I drank fruit drinks 2 times yesterday.

O Yes, I drank fruit drinks 3 or more times yesterday.

**18. Yesterday, did you drink any sports drinks?**


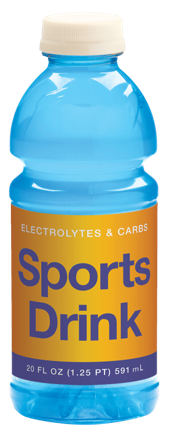

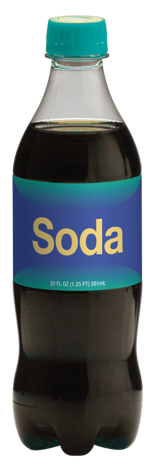

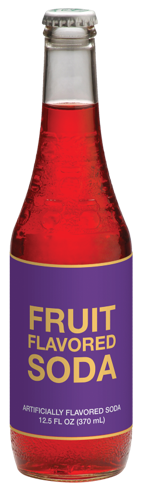

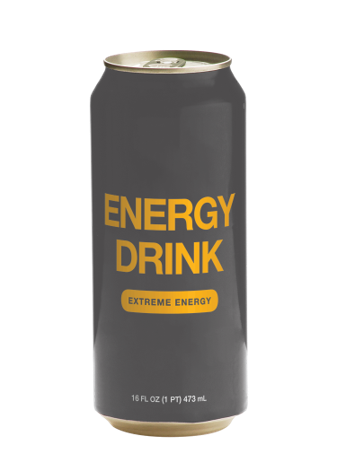


O No, I didn’t drink any sports drinks yesterday.

O Yes, I drank sports drinks 1 time yesterday.

O Yes, I drank sports drinks 2 times yesterday.

O Yes, I drank sports drinks 3 or more times yesterday.

**19. Yesterday, did you drink any regular soda like:**

Cola Root beer

Lemon-lime soda Other regular (non-diet) soda

O No, I didn’t drink any regular soda yesterday.

O Yes, I drank regular soda 1 time yesterday.

O Yes, I drank regular soda 2 times yesterday.

O Yes, I drank regular soda 3 or more times yesterday.

**20. Yesterday, did you drink any energy drinks?**

O No, I didn’t drink any energy drinks yesterday.

O Yes, I drank energy drinks 1 time yesterday.

O Yes, I drank energy drinks 2 times yesterday.

O Yes, I drank energy drinks 3 or more times yesterday.


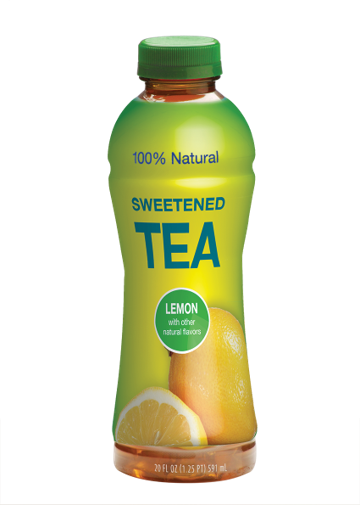
**21. Yesterday, did you drink any sweetened coffee or tea drinks like:**

Frappé Chai

Milk or Boba tea Other sweetened coffee or tea drinks

**Do not include unsweetened coffee or tea.**

O No, I didn’t drink any sweetened coffee or tea drinks yesterday.

O Yes, I drank sweetened coffee or tea drinks 1 time yesterday.

O Yes, I drank sweetened coffee or tea drinks 2 times yesterday.

O Yes, I drank sweetened coffee or tea drinks 3 or more times yesterday.

**22. Yesterday, did you drink any flavored milk or milk-type drinks like:**


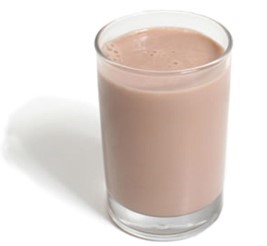
 Chocolate or strawberry milk Yogurt drinks

Flavored rice, almond, or soymilk Horchata

Hot chocolate Sweetened smoothies

Other flavored milk or milk-type drinks

O No, I didn’t drink any flavored milk or milk-type drinks yesterday.

O Yes, I drank flavored milk or milk-type drinks 1 time yesterday.

O Yes, I drank flavored milk or milk-type drinks 2 times yesterday.

O Yes, I drank flavored milk or milk-type drinks 3 or more times yesterday.


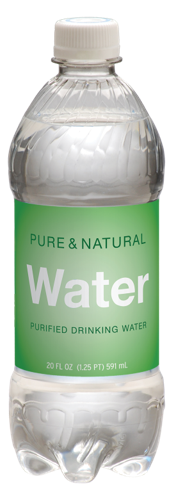
**23. Yesterday, did you drink any water like:**

Tap water Unsweetened sparkling water

Bottled water Water from a fountain

Other unsweetened water

O No, I didn’t drink any water yesterday.

O Yes, I drank water 1 time yesterday.

O Yes, I drank water 2 times yesterday.

O Yes, I drank water 3 or more times yesterday.

The next questions are about your **physical activity.**

**24. Last week, on which days were you physically active for a total of at least 60 minutes**

**(1 hour) per day?**

Add up all the time you spent in any kind of physical activity that made your heart beat fast and

made you breathe hard. Examples: basketball, soccer, running or jogging, dancing, swimming,

tennis, or bicycling.

**
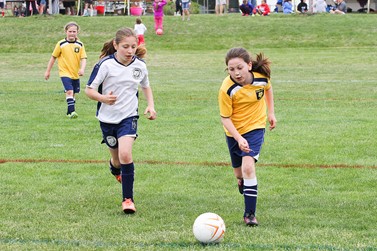
 Choose all that apply.**

□ Monday

□ Tuesday

□ Wednesday

□ Thursday

□ Friday

□ Saturday

□ Sunday

□ I did not do any exercise last week that made my heart beat fast for at least 60 minutes

**25. Last week, on which days did you attend school in person?**

**Choose all that apply.**

□ Monday

□ Tuesday

□ Wednesday

□ Thursday

□ Friday

□ I did not attend school in person last week

**26. Last week…**

O I had recess **every day** I was at school

O I had recess **some days** I was at school

O I did not have recess at school

O I did not have school in person

**27. Last week, when I had recess at school, I did physical activities like:**


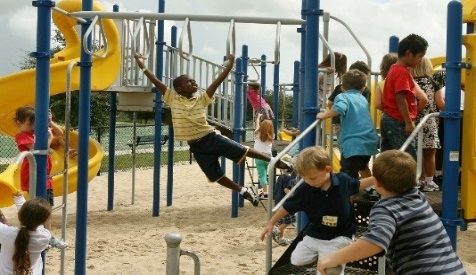
 Sports Playing actively with friends

Physically active games Other activities that got your body moving

O **Most** of the time

O **Some** of the time

O **None** of the time

O I did not have recess last week

O I did not have school in person last week

The next two questions ask about any physical activity classes like PE that you had during school last week. Include classes that you had at school or as part of distance learning. **Do not include activities outside of school like dance class, sports leagues, or martial arts**.

**28. Last week, when did you have a physical activity class like PE?**

**(At school or as part of distance learning).**

**Choose all that apply.**

□ Monday

□ Tuesday

□ Wednesday

□ Thursday

□ Friday

□ I did not have a physical activity class like PE last week

**29.** **Last week, when you had a physical activity class like PE, how much time did you spend**

**doing physical activities like:**

Sports Dancing

Physically active games Other activities that got your body moving


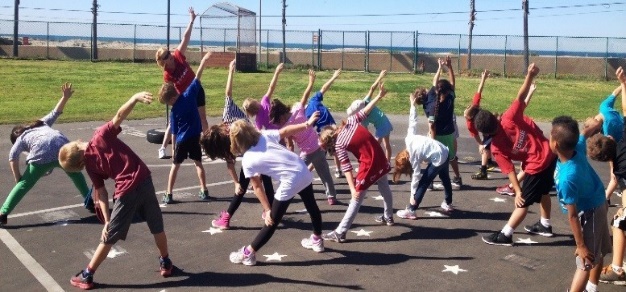


O **Less than half** of the class time

O **About half** of the class time

O **Most or all** of the class time

O I did not have a physical activity class like PE last week

**This is the end of the survey.**

**Thank you!**
